# Associations between Sex Hormones and Circulating Growth Differentiation Factor-15 in Male Patients with Major Depressive Disorder

**DOI:** 10.3390/brainsci11121612

**Published:** 2021-12-07

**Authors:** Ting Sun, Rui Peng, Xiaojun Sun, Yan Li

**Affiliations:** 1 Department of Clinical Laboratory, Renmin Hospital of Wuhan University, Wuhan 430060, China; lejuan0323@whu.edu.cn (T.S.); pengruiwec@163.com (R.P.); 2 Department of Stomatology, Taihe Hospital, Hubei University of Medicine, Shiyan 442000, China; sxj960920@163.com

**Keywords:** sex hormones, depression, growth differentiation factor-15

## Abstract

The interaction between the endocrine system and inflammation is crucial pathogenesis of depression. Our study aimed at exploring the possible relationship between sex hormones and growth differentiation factor-15 (GDF-15), a common indicator of inflammation in male patients with major depressive disorder (MDD). Methods: GDF-15 levels of 121 male MDD patients were compared with 105 healthy subjects with the help of a Cobas 8000 automatic chemiluminescence immunoanalyzer. Results: (1) MDD patients showed higher GDF-15 levels, a lower testosterone (T) level and testosterone/estradiol ratio (T/E2 ratio) than healthy subjects (all *p* < 0.05). (2) Serum T levels and the T/E2 ratio were inversely associated with GDF-15 serum levels (all *p* < 0.05). (3) HAMD-24 scores were positively correlated with the levels of GDF-15 (*p* < 0.01), but not with T levels, estradiol (E2) levels, and the T/E2 ratio (all *p* > 0.05). Conclusion: The high level of GDF-15 was correlated with a low T/E2 ratio and T deficiency in male MDD patients. The above results demonstrate that up-regulation of serum GDF-15 and down-regulation of T and T/E2 ratio may be correlated with the occurrence and severity of depression. So, changing the level of GDF-15 by regulating the proportion of sex hormones may play a key role in the prognosis and treatment of depression.

## 1. Introduction

Major depressive disorder (MDD) is a serious and complex psychiatric disorder with a high rate of disability, morbidity, and recurrence, which may seriously damage social and cognitive functions [1,2,3]. MDD is projected to become the leading cause of death and disability by 2030 [4,5]. Therefore, it is critical to explore the modifying risk factors and effective prevention methods of depression.

In the last few decades, promising research regarding the pathogenesis and etiology of MDD has been underway. Nevertheless, MDD is still not fully understood up. In recent years, research has indicated that changes in sex hormone levels are closely related to depressive symptoms [6,7]. Numerous clinical studies have demonstrated that androgen has a protective effect on the development of affective disorders—a finding that may contribute to improving the depressive symptoms of male and female patients [6,8]. Androgen increases dopamine production in the mesencephalic limbic system, which can prevent the decrease of dopamine activity in the brain and the associated depression-induced loss of pleasure [9,10]. A study of male subjects suffering from hypogonadism has shown that decreased testosterone (T) increases the prevalence of depression in patients with hypogonadism [11] and that T supplementation was able to significantly improve their depression and anxiety symptoms [12,13]. However, in a different study, T replacement therapy showed no significant improvement in depressive symptoms in androgen deficient men compared to placebo-treated controls [14]. One limitation found in the previous work on the relationship between sex hormone levels and depression is that it assessed the estradiol (E2) and T effects independently; thus, the potential interactions or synergistic effects between the two were overlooked. Another weakness is that most previous studies failed to adjust for confounding factors that may affect T levels and depressive symptoms. In fact, T can enzymatically be converted to E2 by aromatase, rendering the production of E2 dependent on aromatase and circulating T [15,16]. Our group previously demonstrated that an unbalanced T/E2 ratio is closely related to the etiology and pathogenesis of MDD (which is mediated by the inflammatory response) and that a combined T and E2 supplementation is more effective than T or E2 alone [8].

Evidently, depression is a mental disease that is closely related to inflammation. Findings on depression in patients who displayed increased levels of peripheral inflammatory factors such as IL-6 [17,18], CRP [19], and TNF-α [20] can confirm this. At the same time, robust empirical evidence suggested that high proinflammatory cytokines levels can predict the risk of MDD development [21]. GDF-15, an important inflammatory factor, has been extensively explored in recent years. In the physiological environment, the levels of GDF-15 are low but are significantly up-regulated in a malignant tumor, ischemia, hypoxia, and inflammatory diseases, indicating that it may play a major role in stress induction [22]. Multiple sources have suggested that GDF-15 can accelerate the progression of neuropsychiatric disorders and that it is closely related to cognitive dysfunction [23,24]. Meanwhile, growing research has shown the value of serum GDF-15 in augmenting the diagnosis of depression and proved that high GDF-15 levels increase the mortality of depression, which may affect the progress and prognosis of depression [25,26]. Consequently, with growing evidence, a relationship between progress and prognosis of depression and the serum GDF-15 levels has become generally accepted. However, it is not yet well supported in relation to other biomarkers. Thus, clarification of this relationship is worth pursuing further since GDF-15 could emerge as a valuable biomarker for the evaluation of depression.

Moreover, GDF-15 can be directly regulated by sex hormones in some tumor cells, which indicates that there might be a relationship between serum sex hormone levels and GDF-15 levels [16,27]. Our study aimed at evaluating the possible link between sex hormones and GDF-15 in male MDD patients with a particular emphasis on E2 and T.

## 2. Methods

### 2.1. Research Objects

All male MDD patients were recruited consecutively in the clinical psychology and psychiatric department of Renmin Hospital of Wuhan University from February 2020 to April 2021. In total, 226 participants, including 121 depression patients (aged 28 (18–41.5)) and 105 healthy controls (aged 32 (26–35)), were identified as being potentially eligible for the present study. The inclusion criteria for healthy controls comprised no family history of mental illness or traumatic life events, tumors, kidney or heart diseases, diabetes, trauma, acute or chronic infection, nor any other diagnosed chronic diseases. The diagnosis of MDD and the evaluation of the severity of depressive symptoms were carried out according to the International Classification of Diseases-10 criteria [2] and the 24-item Hamilton Depression Scale (HAMD-24) [28]. Male MDD patients suffering from CNS, severe head trauma, and inflammation and those taking antidepressants or other antipsychotic medications were excluded. The study was supported by the Medical Ethics Review Committee of Renmin Hospital at Wuhan University (No. WDRY2021-K041). All subjects signed a written informed consent before inclusion into the study.

### 2.2. Sample Preparation

The subjects fasted for at least 8 h prior to the examination and for analysis, and 3 mL venous blood was drawn from the elbow on the morning of the examination. Serum specimens from all subjects were collected by centrifugation at 3500× *g* for 10 min and stored at −70 °C until analysis.

### 2.3. Laboratory Analyses

GDF-15 serum levels were measured by Cobas 8000 automatic chemiluminescence immunoanalyzer (Roche, Ibaraki-Ken, Japan) (Reagent batch No.: 48277001, Cobas, Germany). All serum samples were tested for creatinine (Cr), glucose (Glu), Urea, high-sensitivity C-reactive protein (hs-CRP), uric acid (UA), total cholesterol (TC), and all other blood lipids by an Advia 2400 automatic biochemistry analyzer (Siemens, Erlangen, Germany). Serum E2 and T levels were detected with the help of an ADVIA Centaur CP instrument of Siemens; White blood cell (WBC) count was determined using the Sysmex XN-20 system in Japan.

### 2.4. Statistical Analysis

Analyses were performed using Graphpad Prism 7.0 and SPSS 22.0. LDL-C, TC, UA, and TC/HDL-C were expressed as mean ± SD and compared using an independent sample *t*-test. Non-normally distributed continuous data were represented by a median (interquartile range, IQR) and compared using the Mann–Whitney U test. Categorical variables (expressed in %) were compared by the chi-square test. Multiple linear regression and Spearman correlation analysis were used to further explore the association between variables.

## 3. Results

### 3.1. Study Population

MDD patients had lower levels of HDL-C (high-density lipoprotein cholesterol), Cr, TC, Glu, and LDL-C (low-density lipoprotein cholesterol) compared to healthy subjects (all *p* < 0.01). In addition, levels of inflammatory indexes such as hs-CRP and WBC were higher in male MDD patients than in healthy subjects (both *p* < 0.05). The level of GDF-15 increased, and the level of serum T and the T/E2 ratio decreased in male MDD patients (all *p* < 0.05). The differences of other variables between the two groups were not comparable (Table 1).

### 3.2. Correlation between Sex Hormone Levels and GDF-15

**Figure 1:**
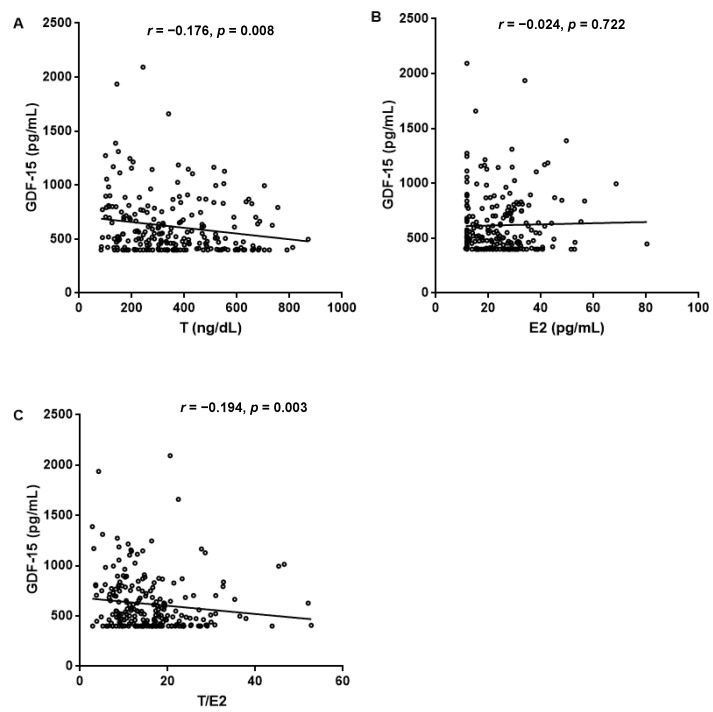
Spearman correlation between sex hormone and GDF-15 levels.

Spearman correlation analysis indicated that serum GDF-15 levels were negatively associated with T levels (*r* = −0.176), and the T/E2 ratio (*r* = −0.194) (both *p* < 0.01) (Table 2) (Figure 1).

### 3.3. Association of HAMD-24 Scores with Sex Hormone and GDF-15 Levels

**Figure 2:**
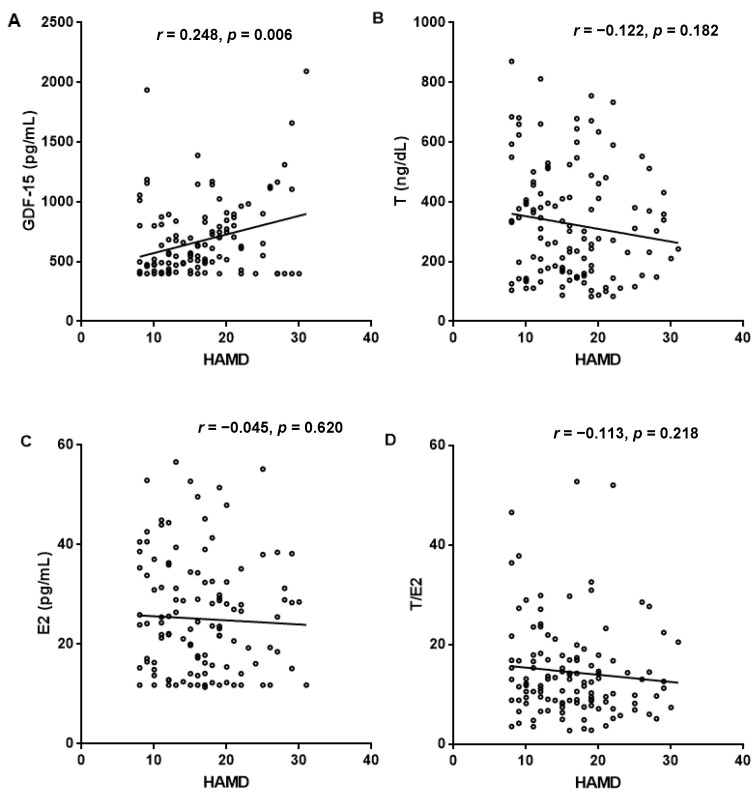
Spearman correlation of sex hormone and circulating GDF-15 levels with HAMD-24 scores in MDD patients.

To further explore the correlations between sex hormone and GDF-15 levels and the severity of depression, multivariate linear regression and Spearman correlation analysis were performed. As shown in Table 3 and Figure 2, after an adjustment for family history of depression, smoking, age, and traumatic life events, HAMD-24 scores were positively correlated with GDF-15 serum levels (β-coefficient = 0.248, *p* < 0.01) but not with T levels (β-coefficient = −0.141), E2 levels (β-coefficient = −0.023), and the T/E2 ratio (β-coefficient = −0.125) (all *p* > 0.05).

### 3.4. Correlation between GDF-15 and Risk Factors of Depression

We performed a Spearman correlation analysis to explore the possible link between GDF-15 serum levels and other independent risk factors of depression in male MDD patients. We could show that GDF-15 was positively associated with Urea, Cr, hs-CRP, Glu, TC, and TG (*r* = 0.187, 0.259, 0.398, 0.206, 0.184 and 0.243, respectively; *p* < 0.05, (Table 4)).

## 4. Discussion

According to these study results, male MDD patients had higher serum GDF-15 levels, lower T levels, and a lower T/E2 ratio than healthy subjects. Furthermore, serum GDF-15 levels were inversely related to T levels and the T/E2 ratio but positively correlated with HAMD-24 scores. In addition, we could show that the TC level of depressed patients is lower than that of the controls, which contradicts to results of most studies performed so far. We hypothesize that this phenomenon might be related to the patients’ dietary status and suicidal ideation since low TC levels are risk factors for the latter [29].

GDF-15 plays a vital role in apoptosis and acute and chronic inflammatory response, and its overexpression modulates a variety of cellular functions and biological processes [30,31]. In addition, similarly to previous reports, we found that depression, a common neurodegenerative disorder, is also strongly correlated with the level of serum GDF-15. Moreover, up-regulated GDF-15 levels can increase the inflammatory response, and both, inflammation and oxidative stress act synergistically, rendering the pathogenesis of depression more complex [32]. Furthermore, GDF-15 has also been related to endothelial injury [33] and dysfunction [34], which play a crucial role in the pathogenesis and treatment of MDD [35]. A study enrolling 310 Chinese patients that experienced an ischemic stroke reported that GDF-15 exacerbated the development of post stroke depression [26]. A similar study of 478 elderly Dutch people by Teunissen et al. [36] indicated that, although GDF-15 was not an independent biomarker of advanced inflammation, the higher the level of GDF-15, the higher the risk of depression. Consistent with the above results, our findings support the view that the HAMD-24 score was positively associated with the level of GDF-15.

Numerous studies have illustrated a robust association between serum GDF-15 levels and serum sex hormone levels [37,38]. The anti-inflammatory effects of sex hormones are well known, and GDF-15, as an essential inflammatory factor, may therefore be affected by sex hormone levels. Miyaue et al. [39] proved that GDF-15 serum levels of male patients with Parkinson’s disease were higher than those of female patients, suggesting significant gender differences in GDF-15 serum levels. Moreover, in prostate cancer cells, the upregulation of GDF-15 was directly affected by the downregulation of T and E2 [40]. Studies have shown that GDF-15 serum levels were negatively associated with T levels and the T/E2 ratio, and the combination of T and E2 could significantly reduce the GDF-15 level [41,42]. Based on the currently available literature, it can therefore be assumed that there is a regulatory interaction between serum GDF-15 and sex hormones.

In addition, numerous previous studies have offered meaningful insights into the role that sex hormones play in depression. It was found that the disorders of sex hormone level regulations, especially T and E2 levels, are closely related to depression. Notably, our results suggest that men suffering from depression have lower serum T levels and a lower T/E2 ratio, which would lead to the conclusion that T has a neuroprotective effect, at least to some extent. Our previous studies indicated that the T/E2 ratio is fixed in women and men and that T and E2 can protect the cardiovascular and nervous system through its anti-inflammatory effects and may, consequently, be involved in the development of MDD [8,41]. Thus, disturbed sex hormones may further contribute to the change of GDF-15, ultimately leading to depression.

The import of our findings notwithstanding, it is necessary to consider some of their limitations. Above all, the sample size of our study was not sufficient, resulting in highly variable GDF-15 levels. Furthermore, the enrolled study patients were of limited geographical distribution. Finally, our cross-section of retrospective studies is limited, which is why our findings can only indicate correlations and not causality.

In conclusion, our study indicates that serum GDF-15 levels are related to sex hormone levels and accompanying depressive symptoms, shedding new light on our understanding of depression. Inhibiting the deleterious effects of GDF-15 in neuropsychiatric disorders by balancing the T/E2 ratio may be a novel strategy for the treatment of depression.

## Figures and Tables

**Table 1:** Clinical characteristics of male MDD patients and controls.

Variable	Controls (n = 105)	MDD Patients (n = 121)	Statistics	*p*
Clinical variables				
Age (years)	32.00 (26.00–35.00)	28.00 (18.00–41.50)	*Z* = −1.600	0.109
Smoking n (%)	12 (11.43%)	17 (14.05%)	*χ*^2^ = 0.345	0.557
Alcohol consumption n (%)	7 (6.67%)	11 (9.09%)	*χ*^2^ = 0.451	0.502
Traumatic life events n (%)	-	6 (4.96%)	-	-
Family history of depression n (%)	-	17 (14.05%)	-	-
HAMD-24 score	-	16 (11.50–20.00)	-	-
Laboratory variables				
Urea(mmol/L)	4.70 (4.12–5.50)	4.62 (3.77–5.40)	*Z* = −1.416	0.157
UA(µmol/L)	398.70 ± 81.39	391.62 ± 93.57	*t* = −0.603	0.547
Glu (mmol/L)	4.68 (4.28–5.09)	4.18 (3.90–4.52)	*Z* = −6.391	<0.001
Cr(µmol/L)	72.00 (66.00–80.00)	66.00 (62.00–75.00)	*Z* = −4.077	<0.001
TC (mmol/L)	4.48 ± 0.64	3.89 ± 0.76	*t* = −6.386	<0.001
WBC (10^9^/L)	5.72 (4.86–6.40)	6.09 (5.18–7.00)	*Z* = −2.759	0.006
HDL-C (mmol/L)	1.09 (0.98–1.29)	1.01 (0.89–1.17)	*Z* = −3.442	0.001
TG (mmol/L)	1.15 (0.92–1.59)	1.16 (0.82–1.66)	*Z* = −0.408	0.683
hs-CRP (mg/L)	0.11 (0.03–0.42)	0.18 (0.04–0.91)	*Z* = −2.117	0.030
LDL-C (mmol/L)	2.66 ± 0.56	2.21 ± 0.70	*t* = −5.404	<0.001
sdLDL-C (mmol/L)	0.72 (0.56–0.93)	0.62 (0.49–0.92)	*Z* = −1.828	0.068
TC/HDL-C	4.04 ± 0.84	3.87 ± 1.04	*t* = −1.338	0.182
T (ng/dL)	355.59(261.63–482.73)	279.12 (157.17–443.73)	*Z* = −2.891	0.004
E2 (pg/mL)	22.05 (15.32–28.75)	23.61 (14.11–32.59)	*Z* = −0.905	0.365
T/E2 ratio	17.22 (12.18–21.14)	12.44 (8.43–17.17)	*Z* = −4.299	<0.001
GDF-15 (pg/mL)	478.00 (405.50–649.50)	551.00(419.50–806.00)	*Z* = −2.408	0.016

HAMD-24: 24-item Hamilton Depression Scale; GDF-15: serum growth differentiation factor-15; MDD: Major depressive disorder; sdLDL-C: small dense low-density lipoprotein cholesterol; Cr: creatinine; Glu: glucose; T: testosterone; HDL-C: high-density lipoprotein cholesterol; TG: triglyceride; UA: uric acid; hs-CRP: high-sensitivity C-reactive protein; WBC: white blood cell; E2: estradiol; LDL-C: low-density lipoprotein cholesterol; T/E2 ratio: testosterone/estradiol ratio; TC: total cholesterol.

**Table 2:** Correlation between sex hormone and GDF-15 levels.

	T	E2	T/E2 Ratio
*r*	−0.176	−0.024	−0.194
*p*	0.008	0.722	0.003

GDF-15: growth differentiation factor-15; T: testosterone; E2: estradiol; T/E2 ratio: testosterone/estradiol ratio.

**Table 3:** Multivariate linear regression to identify associations of sex hormone and GDF-15 levels with HAMD-24 scores in MDD patients.

	(Constant)	GDF-15	T	E2	T/E2 Ratio
Standardized β-coefficient ^a^		0.248	−0.141	−0.023	−0.125
*t*	2.646	2.737	−1.507	−0.249	−1.331
*p*	0.009	0.007	0.134	0.804	0.186

^a^, dependent variable (HAMD-24 scores) (log-transformed). After adjustment for age (log-transformed), alcohol consumption, smoking, traumatic life events and family history of depression. T (log-transformed): testosterone; E2 (log-transformed): estradiol; T/E2 ratio: testosterone/estradiol ratio; GDF-15 (log-transformed): growth differentiation factor-15.

**Table 4:** Spearman correlation coefficients between circulating GDF-15 and risk factors of depression in MDD patients.

	Urea	Cr	UA	hs-CRP	WBC	Glu	TC	TG	HDL-C	LDL-C	sdLDL-C	TC/HDL-C
*r*	0.187	0.259	−0.040	0.398	0.024	0.206	0.184	0.243	−0.023	0.129	0.165	0.154
*p*	0.040	0.004	0.660	<0.001	0.793	0.024	0.043	0.007	0.803	0.158	0.071	0.092

MDD: Major depressive disorder; sdLDL-C: small dense low-density lipoprotein cholesterol; WBC: white blood cell; Glu: glucose; TC: total cholesterol; hs-CRP: high-sensitivity C-reactive protein; TG: triglyceride; UA: uric acid; HDL-C: high-density lipoprotein cholesterol; GDF-15: serum growth differentiation factor-15; Cr: creatinine; LDL-C: low-density lipoprotein cholesterol.

## Data Availability

The datasets used during the current study can be obtained from the corresponding author on reasonable request.
